# Prognostic impact of PCR-based identification of isolated tumour cells in the peritoneal lavage fluid of gastric cancer patients who underwent a curative R0 resection

**DOI:** 10.1038/sj.bjc.6603909

**Published:** 2007-07-31

**Authors:** K Katsuragi, M Yashiro, T Sawada, H Osaka, M Ohira, K Hirakawa

**Affiliations:** 1 Department of Surgical Oncology, Osaka City University Graduate School of Medicine, 1-4-3 Asahi-machi, Abeno-ku, Osaka 545-8585, Japan

**Keywords:** gastric cancer, micrometastasis, prognosis, peritoneal dissemination

## Abstract

Identification of cancer cells in the peritoneal cavity could influence therapy and outcome of gastric carcinoma patients. The objective of this study was to evaluate the clinical impact of the real-time quantitative polymerase chain reaction-(PCR) based identification of isolated tumour cells in the peritoneal lavage fluid of gastric carcinoma. The peritoneal lavage fluid of 116 patients with gastric cancer was sampled at laparotomy. After RNA extraction and reverse transcription, real-time quantitative PCR was performed using the primers and probes for *carcinoembryonic antigen (CEA) and cytokeratin-20 (CK20).* When either the *CEA* mRNA or *CK20* mRNA level of the sample was over the cutoff value, the sample was determined to be PCR-positive. Forty-six (40%) of the 116 patients were PCR-positive and 30 (65%) of the 46 PCR-positive patients died as a result of recurrent peritoneal dissemination. The prognosis of the 46 PCR-positive patients was significantly (*P*<0.001) worse than that of 70 PCR-negative patients. Furthermore, in 80 of the cases with a curative R0 resection, 15 of the patients with PCR-positive findings had a significantly (*P*<0.001) poorer prognosis than the 65 PCR-negative patients. The prognosis of the PCR-positive patients was significantly poorer than that of the PCR-negative patients in the T3 (*P*<0.0001) and T4 (*P*=0.048) subgroups. In a multivariate analysis of the 80 cases with a curative R0 resection, the real-time quantitative RT–PCR (CEA and/or CK20) levels indicated that they were independent prognostic factors. The real-time quantitative RT–PCR analysis of the CEA and/or CK20 transcripts in the peritoneal lavage fluid is useful for predicting the peritoneal recurrence in patients who are undergoing a curative resection for gastric cancer.

The prognosis of patients with early gastric cancer has been improved in recent years. However, patients with an advanced form of gastric carcinoma, especially a serosa-invading tumour such as a T3 or T4 cancer, still have a poor prognosis (Martin *et al*, 2002; Tsuburaya *et al*, 2005). The detection of micrometastases and disseminated cancer cells in patients with tumours who are undergoing curative surgery is a challenging field in oncology because the dissemination of neoplastic cells is the main reason for distant relapse and cancer-related death (Timar *et al*, 2001). Peritoneal dissemination is the most common type of recurrence after surgery in these advanced cases and is one of the reasons for their poor prognosis (Baba *et al*, 1989; Yoo *et al*, 2000). Peritoneal dissemination may arise from the free cancer cells in the peritoneal cavity exfoliated mainly from the serosal surface of the primary tumour. Cancer identification of the tumour cells in the peritoneal cavity could influence the therapy and the outcome of the patients undergoing surgery for advanced gastric carcinoma.

Peritoneal lavage cytology at laparotomy has been a standard method for the detection of free tumour cells and a useful predictor of peritoneal recurrence in gastric cancer. However, by the conventional cytology, patients with negative cancer cells have occasionally developed recurrent peritoneal disease after surgery, which thus resulted in a low sensitivity of the cytology (Boku *et al*, 1990; Abe *et al*, 1995; Schofield *et al*, 1997; de Manzoni *et al*, 2006). Almost half of all patients with serosa-invading gastric carcinoma experience peritoneal recurrence even after a curative surgical resection (Martin *et al*, 2002; Tsuburaya *et al*, 2005). These patients with recurrent peritoneal disease have already developed micrometastasis on the peritoneum at the time of surgery. Micrometastases are defined as single disseminated tumour cells or small clusters of neoplastic cells, which can be occasionally detected by conventional cytology (Singletary *et al*, 2003; Sobin, 2003). Recently, the reverse transcriptase–polymerase chain reaction (RT–PCR) technique has made it possible to detect only a few cancer cells in the abdominal cavity and the technique is more sensitive than with traditional peritoneal lavage cytology (Kodera *et al*, 1998; Timar *et al*, 2002). The conventional RT–PCR used for the detection of the proper band of tumour nucleotides on agarose gel is sensitive, but does not always have a high specificity due to the fact that false positives are sometimes obtained as a result of the weak expression in non-cancerous cells, such as mesothelial cells and lymphocytes (Kodera *et al*, 1998; Sakakura *et al*, 2001). The real-time quantitative RT–PCR is a more specific and quantitative method for the detection of tumour nucleotides by free cancer cells in peritoneal washes (Nakanishi *et al*, 2000; Osaka *et al*, 2004). The objective of this study was to clarify the clinical impact of the real-time quantitative RT–PCR-based identification of isolated tumour cells in the peritoneal lavage fluid of gastric carcinoma.

## MATERIALS AND METHODS

### Patients

In this study, 124 patients with gastric cancer were enrolled. A laparotomy and preoperative peritoneal lavage were performed on all 124 patients who intended to undergo a gastrectomy in our department. During the operation, the abdominal cavity was thoroughly examined for tumour metastasis. When a potentially curative resection was considered possible, a gastrectomy with a D2 lymphadenectomy was performed. A palliative resection was performed for the patients who were not treated with a curative resection. A bypass operation was performed for patients who were unable to receive a gastrectomy because of extensive invasion to adjacent organs or extensive peritoneal dissemination. Five patients were excluded who died as a result of liver metastasis (three patients), lung metastasis (one patient), and bone metastasis (one patient). Glyceraldehyde-3-phosphate dehydrogenase (GAPDH) mRNA was undetectable in the samples of three patients. Next, the data from the remaining 116 patients were analysed. Of the 116 patients with resectable cancer, 80 underwent a potentially curative R0 resection, 29 were treated with a palliative resection, and the remaining seven patients were considered unresectable and underwent bypass operations. As negative controls, the peritoneal lavage specimens obtained from 10 of the patients with cholecystolithiasis for cholecystectomy were used. Informed consent to participate in this study was obtained from all of the patients before their surgery. The pathologic diagnoses and classifications were made according to the TNM staging system (Sobin, 2003) of the Union Internationale Contre le Cancer (UICC). After the surgical resection, all patients underwent a follow-up, with the median follow-up at analysis being 32 months for all patients. The follow-up programme of post-operative surveillance consisted of physical examination, blood chemistry including CEA, computed tomography, and ultrasound performed every 3 months to diagnose recurrent diseases.

### Peritoneal lavage sample

Just after laparotomy, 100 ml of normal saline was poured into the Douglas' pouch and then was left in the subphrenic space, and then the peritoneal lavage fluid was collected from the cavity before a surgical resection. Half of the peritoneal lavage fluid was examined through conventional cytological methods with Papanicolaou staining, and for the remaining half, the free cancer cells in the abdominal cavity were detected by means of a molecular diagnosis, as described below.

### RNA extraction

The peritoneal lavage fluid was centrifuged at 2000 r.p.m. for 10 min to collect the intact cells and then dissolved in a TRIZOL Reagent (Gibco, Gaithersburg, MD, USA). The mixture and 0.5 ml of chloroform in a 1.5 ml tube was centrifuged at 12 000 r.p.m. for 15 min. Next, the supernatant was transferred to a fresh tube, and 0.2 ml propanol was added. The RNA was precipitated after centrifugation at 12 000 r.p.m. for 10 min, washed with 75% ethanol, and diluted with DEPC-treated water. The purified RNA was then quantified and assessed for purity by UV spectrophotometry.

### Reverse transcriptase–polymerase chain reaction

Complementary DNA (cDNA) was prepared from each sample using oligo-(dT)_15_ primer and M-MLV Reverse Transcriptase (Life Technologies, Rockville, MD, USA). RT reaction was performed at 37°C for 50 min, followed by heating at 70°C for 10 min. PCR amplification was performed using the following primer sequences, For *cytokeratin20 (CK20)*: forward 5′-CTCTCCTCAAAAAGGAGCATCAG-3′; reverse 5′-CAACCTCCACATTGACAGTGTTG-3′; Taqman probe FAM-CAGATGCTTGTGTAGGCCATCGACTTCCT-TAMRA. For *carcinoembryonic antigen* (*CEA*): forward 5′-CAATAGGACCACAGTCACGACGAT-3′, reverse 5′-GGTTGGAGTTGTTGCTGGTGAT-3′; Taqman probe FAM-ACAGTCTATGCAGAGCCACCCAAACCCTT-TAMRA. For *GAPDH*: forward 5′-GAAGGTGAAGGTCGGAGTC-3′, reverse 5′-GAAGATGGTGATGGGATTTC-3′, Taqman probe VIC-CAAGCTTCCCGTTCTCAGCC-TAMRA. Real-time quantitative RT–PCR was carried out using the ABI PRISM 7700 Sequence Detection system (Applied Biosystems, Foster City, CA, USA) per the manufacturer's protocol. The total volume of the PCR was 50 *μ*l, containing 2 *μ*l of cDNA template, 25 *μ*l TaqMan Universal PCR Master Mix (Applied Biosystems), 0.1 *μ*M probe, and 0.3 *μ*M of each primer. The PCR conditions were as follows: after incubation at 50°C for 2 min and denaturing at 95°C for 10 min, 40 cycles of 15 s at 95°C, and 60 s at 60°C. The mRNA level of each gene was normalised by the internal control *GAPDH*.

### Standard curve

The gastric cancer cell line, OCUM-2M (Yashiro *et al*, 1995), was used for the construction of a standard curve of *CEA* and *CK20* expression. The cDNA from 1 *μ*g of RNA from the OCUM-2M cells was diluted with normal monocytes at various ratios. The PCR cycling conditions according to the manufacturer's protocol were 40 cycles of 15 s at 95°C and 60 s at 60°C. The threshold cycle *C*_t_ represents the fractional cycle number at which a significant increase above the baseline signal was first detected. The log starting copy number is plotted against the threshold cycle *C*_t_. The standard curves for *CEA*, *CK20*, and *GAPDH* were then constructed. According to the standard curve, the *CEA* and *CK20* mRNA levels were calculated.

### Statistical analysis

We used the *χ*^2^ test, Fisher's exact test, or the Mann–Whitney *U*-test to determine the significance of the differences between the covariates. The survival durations were calculated using the Kaplan–Meier method and were analysed by the log–rank test to compare the cumulative survival durations in the patient groups. The survival curve was calculated from the date of surgery. The Cox proportional hazards model was used to compute the univariate and multivariate hazards ratios for the study parameters. For all of the tests, a *P-*value of less than 0.05 was considered to be statistically significant. The SPSS software program (SPSS Japan, Tokyo, Japan) was used for the analyses.

## RESULTS

### Determination of cutoff values

**Figure 1:**
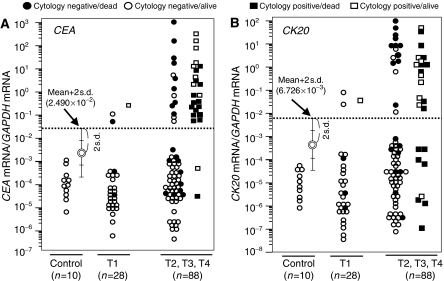
Expression levels of *CK20* and *CEA* mRNA. *CEA/GAPDH* mRNA ratios and *CK20/GAPDH* mRNA ratios were shown according to depth of tumour invasion. The *CEA* mRNA and *CK20* mRNA expression of the peritoneal lavage fluid of the control samples (*n*=10), T1 (*n*=28), T2 (*n*=23), T3 (*n*=52), and T4 (*n*=13), were quantified by RT–PCR. The *CEA/GAPDH* mRNA ratios and *CK20/GAPDH* mRNA ratios were plotted according to T stage of TNM classification. Each cutoff value was determined based on the mean plus 2 s.d. of the *CEA/GAPDH* mRNA ratios and *CK20/GAPDH* mRNA ratios level in the peritoneal lavage fluid of the control and T1 samples. (**A**) The cutoff value of *CEA/GAPDH* was 2.490 × 10^−2^. *CEA* was positive in 3 (11%) of the 28 patients with T1 cancer and in 35 (40%) of the 88 patients with T2, T3, or T4 cancer. (**B**) The cutoff value of *CK20/GAPDH* was 6.726 × 10^−3^. *CK20* was positive in 3 (11%) of the 28 patients with T1 cancer, and 31 (35%) of the 88 patients with T2, T3, or T4 cancer were *CK20*-positive. Sixty-four (73%) of the 88 patients with T2, T3, or T4 cancer were cytology-negative, and 19 (30%) of the 64 cytology-negative patients with T2, T3, or T4 cancer died due to peritoneal recurrence. In these 19 patients, *CEA* or *CK20* was positive in 9 and 11, respectively. The open or closed circles show the cytology-negative patients. The open or closed squares show the cytology-positive patients. The closed circle and closed square show patients who died by peritoneal recurrence Owing to the TNM staging system of the Union Internationale Contre le Cancer.

Figure 1 shows the *CEA/GAPDH* mRNA ratios and the *CK20/GAPDH* mRNA ratios according to the depth of the tumour invasion. Each cutoff value was determined as the mean plus 2 s.d. based on the quantified values of the control and the T1 samples. The cutoff value of the *CEA/GAPDH* (Figure 1A) and *CK20/GAPDH* (Figure 1B) levels were 2.490 × 10^−2^ and 6.726 × 10^−3^, respectively. When the *CEA* or *CK20* mRNA/*GAPDH* mRNA level of the sample was above the cutoff value, the mRNA expression was considered positive, and the sample was determined to be PCR-positive.

### Correlation between clinicopathological factors and *CEA and/or CK20* expression in the peritoneal lavage fluid

All 116 patients were subjected to both a *CEA and/or CK20* examination and a cytological examination. All of the peritoneal lavages from the control (*n*=10) were negative for the quantitative RT–PCR of *CEA* and/or *CK20* expression. *CEA* was positive in three (11%) of the 28 patients with T1 cancer and in 35 (40%) of the 88 patients with T2, T3, or T4 cancer (Figure 1A). *CK20* was positive in three (11%) of the 28 patients with T1 cancer, and 31 (35%) of the 88 patients with T2, T3, or T4 cancer were *CK20*-positive (Figure 1B). Sixty-four (73%) of the 88 patients with T2, T3, or T4 cancer were cytology-negative, and 19 (30%) of the 64 cytology-negative patients with T2, T3, or T4 cancer died as a result of peritoneal recurrence. In these 19 patients, *CEA* or *CK20* was positive in 9 and 11, respectively (Figure 1). Table 1 summarises the correlation between the CEA and/or CK-20 expression of the peritoneal lavage fluid and the clinicopathological parameters. The molecular diagnosis significantly correlated with the T stage as the depth of tumour invasion (*P*<0.001), peritoneal dissemination at operation (*P*<0.001), cytology (*P*<0.001), stage (*P*<0.001), and lymph node disease (*P*<0.001). In contrast, there was no statistically significant association between *CEA* and/or *CK20* and hepatic metastasis at operation, tumour differentiation, venous invasion, or lymphatic invasion.

### Recurrent peritoneal metastasis

Peritoneal recurrence was analysed in 116 patients. Table 2 summarises the correlation between peritoneal recurrence in these 116 cases and the results of the peritoneal lavage assays. Thirty-eight (33%) of the 116 patients were positive for *CEA* mRNA and 34 (29%) of the 116 patients were positive for *CK-20* mRNA. Forty-six (40%) patients were positive for either marker and were thus determined to be PCR-positive. The sensitivities and specificities were calculated based on the diagnosis of peritoneal metastases during the postoperative surveillance period. Death by recurrent peritoneal metastasis was found in 37 (32%) of the 116 patients. *CEA* or *CK20* mRNA levels are helpful for the prediction of peritoneal recurrence with a sensitivity of 72.7 or 54.6%, and a specificity of 82.7 or 80.3%, respectively (Table 2). In Figure 1, the closed circle and square show patients who died by peritoneal recurrence. Peritoneal recurrence was frequently found in 34 of the 88 advanced gastric cancer (T2, T3, and T4 categories) patients, while it was found in only three of the 28 early gastric cancer (T1 category) patients. Meanwhile, the combination of *CEA* and/or *CK20* mRNA levels had a sensitivity of 86.4% and a specificity of 81.5%.

### Survival

**Figure 2:**
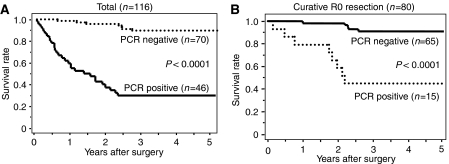
The overall survival of patients based on a PCR-based analysis. The survival curve shows the Kaplan–Meier overall survival curves in relation to the *CEA* and *CK20* mRNA levels in the gastric carcinomas. (**A**) The prognosis of all 116 patients with PCR-positive tumours was significantly (*P*<0.001) worse than that of those with PCR-negative tumours. (**B**) In the 80 patients with a curative R0 resection, the prognosis of the PCR-positive patients (*n*=15) was significantly (*P*<0.001) worse than that of the 65 patients who were PCR-negative.

**Figure 3:**
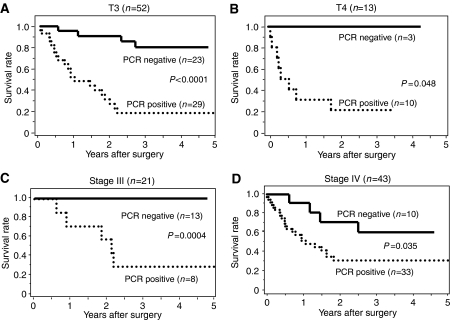
The overall survivals according to the status of T stage or clinical stage. The overall survivals of the subgroups of 116 patients were subdivided according to the status of T stage or clinical stage. The PCR-positive patients had a significantly poor prognosis in comparison to those who were PCR-negative in the pT3 (**A**) and pT4 stages (**B**) The prognosis of the PCR-positive cancer was significantly poorer (*P*=0.026) than that of the PCR-negative cancer in the stage III (**C**) and stage IV (**D**) groups.

The prognosis of all 116 patients with PCR-positive tumours was significantly (*P*<0.001) worse than those with PCR-negative tumours (Figure 2A). Moreover, in the 80 patients with a curative R0 resection, the prognosis of the PCR-positive patients (*n*=15) was significantly (*P*<0.001) worse than those 65 patients who were PCR-negative (Figure 2B). In addition, we analysed the prognostic significance of the *CEA and/or CK20* expression in the four subgroups, T1, T2, T3, and T4 (Figure 3). The prognosis of the patients with PCR-positive cancer was significantly poorer than those with PCR-negative cancer in the T3 (*P*<0.0001; Figure 3A) and T4 (*P*=0.048; Figure 3B) subgroups, while no significant difference in prognosis was found between the *CEA and/or CK20* expression in the T1 and T2 subgroups (data not shown). In the clinical stage, the prognosis of the *PCR-positive* cancer was significantly poorer than the PCR-negative cancer in stage III (*P*=0.0004; Figure 3C) and stage IV (*P*=0.035; Figure 3D), while no significant difference in prognosis was found between the *CEA and/or CK20* expression in stages I and II (data not shown).

We evaluated prognostic markers in 80 patients of curative R0 resection. According to a univariate analysis (Table 3), the RT–PCR (*CEA* and/or *CK20*), T stage, and stage were significantly correlated with patient survival. In a multivariate analysis (Table 4), the RT–PCR (*CEA* and/or *CK20*), and tumour depth were found to be independent prognostic factors.

## DISCUSSION

In patients with a curative R0 resection, the prognosis of the patients with *PCR*-positive tumours was significantly worse than those with PCR-negative tumours. Regarding overall survival, a multivariate analysis showed that the current real-time quantitative RT–PCR technique using *CEA* and/or *CK20* was an independent prognostic factor for the patients with a curative R0 resection. Moreover, the RT–PCR method was found to be useful in T3 or T4 gastric cancer in which peritoneal dissemination is the main cause of death after a surgical resection. These findings suggest that the RT–PCR detection of the CEA and/or CK20 transcripts in the peritoneal lavage specimens has a prognostic relevance in patients who are undergoing a curative resection for T3 and T4 gastric cancer. Most of the current chemotherapy regimens have failed to improve the survival of T3 and T4 gastric cancer patients (Martin *et al*, 2002; Tsuburaya *et al*, 2005). The RT–PCR assay of peritoneal lavage can be a reliable method for the selection of patients for therapeutic chemotherapy.

Many markers, including *CEA*, *CK20*, *CK19*, *MAGE*, and *MMP-7*, have previously been applied to detect micrometastases of gastric cancer (Okami *et al*, 2000; Yonemura *et al*, 2001; Kodera *et al*, 2002; Lin *et al*, 2002; Matsuda *et al*, 2004; Arigami *et al*, 2006; Horibe *et al*, 2007). Our preliminary study showed that the *CK19* and *MMP-7* mRNA were detected in the peritoneal lavage fluid from the control benign disease, and that the *MAGE-1* and *MAGE-3* mRNA were occasionally undetectable in the cytology-positive samples. Also, the *CK19* mRNA was expressed in the normal control tissues, and the presence of the pseudogenes of *CK19* reportedly limits its value. Therefore, *CK19*, *MAGE*, and *MMP-7* were not used as a diagnostic marker for the detection of peritoneal micrometastasis in this study. In contrast, *CEA* has been reported to be a reliable target for the detection of disseminated gastric cancer cells, and is the most common marker for detecting micrometastasis with RT–PCR (Kodera *et al*, 1998; Nakanishi *et al*, 2000; Osaka *et al*, 2004). The quantitative RT–PCR method using multiple markers reportedly improved the sensitivity and specificity of the quantitative RT–PCR method (Guller *et al*, 2002; Timar *et al*, 2002; Osaka *et al*, 2004; Fujita *et al*, 2006). We therefore selected the combination of *CEA* and *CK-20* as diagnostic markers for predicting micrometastasis in patients with gastric carcinoma in this study.

Some papers have reported the use of *GAPDH* mRNA as a quantity marker to possibly not be necessary (Kodera *et al*, 2002; Ito *et al*, 2005). However, *GAPDH* is considered to be necessary because a specific genetic marker for gastric cancer has not yet been found. We used the *CEA/GAPDH* ratio or *CK20/GAPDH* ratio for a subsequent analysis in the present study. The cutoff value was determined based on the mean plus 2 s.d. of the samples of the benign disease and T1 gastric cancers, as well as those of the others (Osaka *et al*, 2004; Oyama *et al*, 2004). The PCR methods resulted in more false positives than other methods. The RT–PCR assay for *CEA* frequently resulted in false negatives (Marutsuka *et al*, 2003) because the expression level of the *CEA* mRNA was heterogeneous in the gastric tumours which exhibited no expression of *CEA* mRNA (Osaka *et al*, 2004), or due to the weak expression in the non-cancerous cells, such as the mesothelial cells. *CK20* also has a relatively low specificity because of the frequent *CK20* expression in not only the cancer cells but also in the normal epithelial cells. In our study, the sensitivity and specificity of the *CEA* or *CK20* RT–PCR assay, which were useful for the prediction of peritoneal recurrence were 64.9 and 82.7% or 54.6 and 80.3%, respectively. The combinations of the CEA and/or CK20 mRNA levels were 86.4 and 81.5%, respectively. The sensitivity of each marker was low, but that in combination with *CK20* and *CEA* increased (81.5%) without any decrease in the specificity. When the cutoff value decreases, then each marker's sensitivity has been observed to increase. The cutoff value in this study is appropriate because both the sensitivity and specificity of multiple markers are high. On the other hand, 6 of 37 of both the cytology-negative and PCR-negative patients died as a result of peritoneal metastasis. The discovery of a novel specific marker for gastric cancer could be expected to create a higher accuracy for the detection of a few cancer cells in the peritoneal lavage fluid.

It has been reported that isolated tumour cells (ITC), which are single tumour cells or a small cell cluster (Sobin, 2003), do not always show morphological evidence of metastatic activity, such as peritoneal metastasis (Cserni *et al*, 2005). The DNA from necrotic cancer cells may be detectable by PCR method in the peritoneal cavity but they are nonviable and therefore their identification at the DNA level would thus be misleading concerning tumour progression. Our study showed that, in the cytology-negative patients, 11 of the 17 PCR-positive patients died due to recurrent peritoneal dissemination, and the prognosis of the PCR-positive patients was significantly (*P*<0.0001) worse than that of the PCR-negative patients. mRNA is detectable in viable cells because RNA is unstable in necrotic cancer cells. These findings suggest that the micrometastatic cells identified at the RNA level by real-time quantitative RT–PCR assay are considered to be viable for developing tumour progression for peritoneal metastasis.

The metastatic processes that are responsible for peritoneal dissemination remain controversial. Tumour cells originating in the abdomen can disseminate to the mesentery in three major ways: extension via the mesenteric lymphatics, embolic haematogenous spread, and intraperitoneal seeding (Meyers *et al*, 1987; Sheth *et al*, 2003; Tendo *et al*, 2006). In this study, the *CEA and/or CK20* examination significantly correlated with the T stage as depth of tumour invasion (*P*<0.001), peritoneal dissemination (*P*<0.001), whereas there was no statistically significant association with venous invasion or lymphatic invasion. These findings suggest that peritoneal dissemination may arise from free cancer cells into the peritoneal cavity exfoliated mainly from the serosal surface of the stomach penetrated by the primary tumour.

In conclusion, a real-time quantitative RT–PCR analysis of the CEA and/or CK20 transcripts in the peritoneal lavage fluid is thus considered to be useful for predicting the peritoneal recurrence in patients who are undergoing a curative resection for gastric cancer. The RT–PCR assay of peritoneal lavage might therefore be a reliable method for the selection of patients who should undergo therapeutic chemotherapy.

## Figures and Tables

**Table 1:** Relationship between the RT–PCR results, cytology and clinicopathological findings at operation in 116 patients with gastric cancer

	**RT–PCR (CEA/CK20)**		
**Clinicopathological findings**	**Positive *n*=46**	**Negative *n*=70**	***P*-value**
*Tumour depth* a			
T1 or T2	7	44	< 0.001
T3 or T4	39	26	
			
*Peritoneal metastasis*			
Positive	12	1	<0.001
Negative	34	69	
			
*Cytology*			
Positive	25	2	<0.001
Negative	21	68	
			
*Stage*			
I or II	5	47	<0.001
III or IV	41	23	
			
*LN metastasis*			
Positive	40	36	< 0.001
Negative	6	34	
			
*Histological type*			
Intestinal type	14	33	0.073
Diffuse type	32	37	
			
*Lymphatic invasion*			
Positive	34	46	0.350
Negative	12	24	
			
*Venous invasion*			
Positive	11	14	0.616
Negative	35	56	
			
*Hepatic metastasis*			
Positive	5	2	0.076
Negative	41	68	

a T1: tumour invades mucosa or submucosa, T2: tumour invades the muscularis propria or the subserosa, T3: tumour penetrates the serosa and exposed to abdominal cavity without invading the adjacent structures, T4: tumour invades the adjacent structures.

Significance level of difference was determined using Fisher's exact test or χ^2^ test.

**Table 2:** Relationship between peritoneal recurrence and *CEA* or *CK20* mRNA expression

**RT-PCR**	**Death (*n*=37)**	**Alive (*n*=79)**	***P*-value**	**Sensitivity (%)**	**Specificity (%)**
*CEA*					
Positive (*n***=**38)	24	14	< 0.001	64.9	82.3
Negative (*n***=**78)	13	65			
					
*CK 20*					
Positive (*n***=**34)	19	15	< 0.001	51.4	81.0
Negative (*n***=**82)	18	64			
					
*CEA and/or CK20*					
Positive (*n***=**46)	30	16	< 0.001	81.1	79.7
Negative (*n***=**70)	7	63			

Sensitivity was defined as the positive rate for the CEA/GAPDH mRNA ratio or the CK20/GAPDH mRNA ratio with peritoneal washes of patients who developed recurrent peritoneal carcinomatosis. Specificity was defined as the negative rate for the CEA/GAPDH mRNA ratio or the CK20/GAPDH mRNA ratio in patients without any signs of peritoneal carcinomatosis.

**Table 3:** Univariate analysis with respect to overall survival in 80 patients of curative R0 resection

**Parameter**	**Risk ratio**	**95% confidence interval**	***P*-value**
*RT—PCR(CEA and/or CK20)*	9.2	3.0–28.5	<0.001
Negative *vs* positive			
			
*Tumour depth*	11.1	2.4–50.1	0.02
T1/T2 *vs* T3/T4			
			
Stage	5.8	1.8–18.8	0.004
1/2 *vs* 3			
			
*Lymph node metastasis*	1.9	0.6–5.7	0.28
Negative *vs* positive			
			
*Lymphatic invasion*	3.1	0.68–13.9	0.144
Negative *vs* positive			
			
*Venous invasion*	2.3	0.72–7.6	0.160
Negative *vs* positive			
			
*Histological type*	0.872	0.29–2.6	0.805
Intestinal type *vs* diffuse type			

**Table 4:** A multivariate analysis regarding the overall survival in 80 patients of curative R0 resection

**Parameter**	**Risk ratio**	**95% confidence interval**	***P*-value**
*RT–PCR(CEA and/or CK20)*	9.9	2.3–43.0	0.002
Negative *vs* positive			
			
*Tumour depth*	14.1	1.7–116.9	0.014
T1/T2 vs. T3/T4			
			
*Stage*	3.1	0.16–62.1	0.458
1/2 *vs* 3			
			
*Lymph node metastasis*	0.2	0.018–2.2	0.19
Negative *vs* positive			
			
*Lymphatic invasion*	0.49	0.073–3.28	0.463
Negative *vs* positive			
			
*Venous invasion*	1.2	0.24–6.0	0.818
Negative *vs* positive			
			
*Histological type*	0.335	0.088–1.27	0.11
Intestinal type *vs* diffuse type			
